# Analyzing brain structural differences among undergraduates with different grades of self-esteem using multiple anatomical brain network

**DOI:** 10.1186/s12938-021-00853-z

**Published:** 2021-02-12

**Authors:** Bo Peng, Gaofeng Pang, Aditya Saxena, Yan Liu, Baohua Hu, Suhong Wang, Yakang Dai

**Affiliations:** 1 Suzhou Institute of Biomedical Engineering and Technology, Chinese Academy of Sciences, Suzhou, China; 2 Suzhou Key Laboratory of Medical and Health Information Technology, Suzhou, China; 3 Jinan Guoke Medical Engineering Technology Development Co., LTD, Jinan, China; 4 Department of Pediatrics, The Third Affiliated Hospital of Soochow University, Changzhou, China; 5 Trauma Center, Khandwa District Hospital, Khandwa, India; 6 Department of Clinical Psychology, The Third Affiliated Hospital of Soochow University, Changzhou, China

**Keywords:** Structural magnetic resonance imaging, Self-esteem, Multi-resolution ROI, Hierarchal brain network, Machine learning method

## Abstract

**Background:**

Self-esteem is the individual evaluation of oneself. People with high self-esteem grade have mental health and can bravely cope with the threats from the environment. With the development of neuroimaging techniques, researches on cognitive neural mechanisms of self-esteem are increased. Existing methods based on brain morphometry and single-layer brain network cannot characterize the subtle structural differences related to self-esteem.

**Method:**

To solve this issue, we proposed a multiple anatomical brain network based on multi-resolution region of interest (ROI) template to study the brain structural connections of self-esteem. The multiple anatomical brain network consists of ROI features and hierarchal brain network features that are extracted from structural MRI. For each layer, we calculated the correlation relationship between pairs of ROIs. In order to solve the high-dimensional problem caused by the large amount of network features, feature selection methods (*t*-test, mRMR, and SVM-RFE) are adopted to reduce the number of features while retaining discriminative information to the maximum extent. Multi-kernel SVM is employed to integrate the various types of features by appropriate weight coefficient.

**Result:**

The experimental results show that the proposed method can improve classification accuracy to 97.26% compared with single-layer brain network.

**Conclusions:**

The proposed method provides a new perspective for the analysis of brain structural differences of self-esteem, which also has potential guiding significance in other researches involved brain cognitive activity and brain disease diagnosis.

## Background

Self-esteem is regarded as self-affirmation and self-identification about oneself [1]. People with good mental health have a higher self-esteem grade and think of themselves as valuable persons [2]. Researchers found that undergraduates with different self-esteem grades have brain structural differences. These people feel that they deserve to be respected by others, and are more able to accept individual's deficiencies [3]. However, people with low self-esteem have low self-confidence, and the outside world will have a great impact on them, resulting in low socioeconomic status and poor physical health. Neurophysiology researchers found that self-esteem may be composed of multiple subsystems that are structurally separated from each other, but functionally interact [4]. Brain imaging studies suggest that self-esteem involves multiple psychological processes, including self in perception, memory, and introspection. These psychological processes have their own corresponding brain regions. For example, self-face recognition occurs in right brain, and autobiographical memory is mainly related to hippocampus, and self-reference is related to medial prefrontal lobe [5]. In addition to these independent brain regions, the difference in self-esteem is also reflected in brain network connection. Medial prefrontal cortex is activated during the process of social, self, and affective events [6]. Therefore, in this study, we focus on exploring the brain structural differences among undergraduates with different levels of self-esteem.

Brain network aims to study the interaction of various brain regions as a whole, which has an important role to deeply understand brain structures and cognitive neural processes. The anatomical brain network mainly uses region of interest (ROI) of the brain as the node, and the correlation between brain regions as edge [7, 8]. The definition of ROI is a key step in anatomical brain network analysis. Most existing methods use ROI-based brain network analysis methods to study brain structure and functional connections related to self-esteem. Kelly et al. use the cerebral blood flow imaging method to estimate the hemodynamic response function of each ROI, in order to study the brain networks that are activated during the processing of self-esteem related information [9]. Goldin et al. used functional magnetic resonance imaging (fMRI) technology to measure changes in the brain network between self-esteem group and the self-confidence group by measuring the BOLD response in the ROI [10]. Chavez et al. conducted a psychophysiological interaction analysis to calculate the correlation between specific ROIs related to self-esteem [11]. Although a variety of neuroimaging methods can be used to explore the cognitive mechanism of the brain, structural magnetic resonance imaging (sMRI) is widely used in the analysis of brain anatomical networks due to its high resolution of brain soft tissue imaging [12]. Studies based on sMRI show that self-esteem involves multiple networks related to self-reference processing, autobiographical memory, and social cognition, including default mode networks and social cognition networks [13]. In addition, self-esteem shows the brain network mechanism dominated by bilateral brain and mainly controlled by right brain [14]. Although the above researches have initially revealed the brain network representation of self-esteem, it only used single-layer network that cannot fully identify the subtle differences in network connectivity caused by self-esteem.

The motivation of this study is to use enhanced feature representation method to better analyze brain structural connectivity related to self-esteem. In recent years, machine learning techniques become a research hotspot in the field of brain network analysis due to its ability to learn pattern from data and predict unknown data [8]. Brain network analysis can help us fully understand the cognitive psychological activity of self-esteem. However, there are few studies using machine learning methods to construct self-esteem-related brain network, especially for the construction of hierarchal brain networks. In this article, we propose multiple anatomical brain network construction method based on multi-resolution ROIs. The innovation of this method is to use the in-layer and between-layer connections to better describe the correlation between small brain regions and large brain functional areas, which improves the feature expression ability of single-layer brain network.

## Results

### Classification performance

**Fig. 1:**
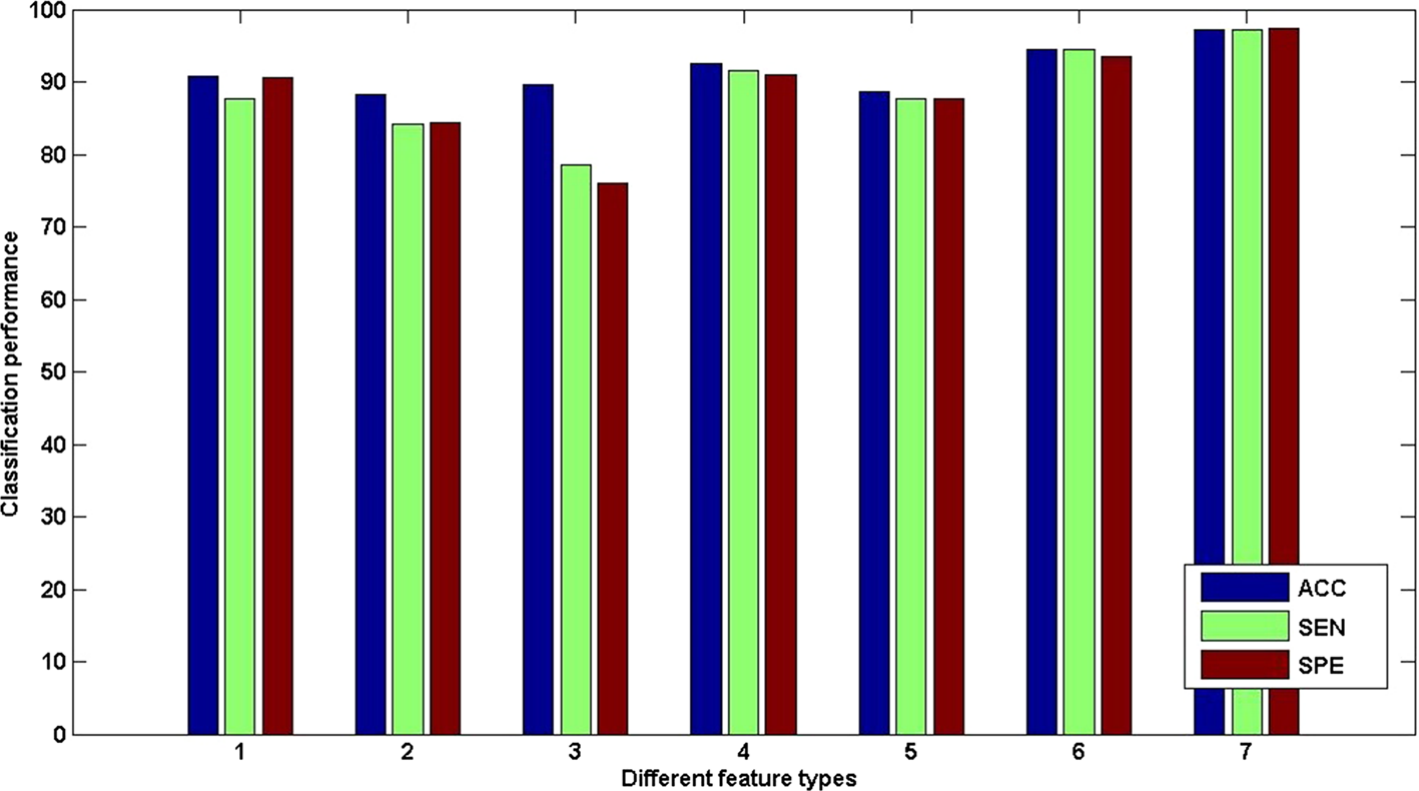
Boxplot of classification accuracy for different feature types. (1) Network features in the 4th layer; (2) network features in the 3rd layer; (3) network features in the 2nd layer; (4) network features in all layers; (5) ROI features in the 4th layer; (6) ROI features and network features in the 4th layer; (7) multilevel features. *ACC* accuracy, *SEN* sensitivity, *SPE* specificity

Various indexes can be used to evaluate the classification performance of the proposed method. The evaluation indicators including accuracy (ACC), sensitivity (SEN), specificity (SPE), area under the receiver operating characteristic curve (AUC), *F* score, balanced accuracy, Youden’s index are listed in Table 1. The results show that the multiple brain network features have the highest classification accuracy of 97.26%, and the AUC is also greater than other feature types. This indicates that the multiple brain network features have advantages in characterizing structural differences at the global level. In addition, the higher specificity and sensitivity also show that the multiple brain network features have better recognition capabilities in exploring the subtle differences in brain structure caused by self-esteem (Fig. 1).

**Table 1:** Classification performance using different feature types

	ACC (%)	AUC (%)	SEN (%)	SPE (%)	*Y* (%)	*F* (%)	BAC (%)
Network features in the 4th layer	90.69	96.63	87.72	90.65	86.74	77.38	88.77
Network features in the 3rd layer	88.31	84.27	85.32	84.29	88.33	82.62	85.74
Network features in the 2nd layer	89.59	76.65	78.53	75.94	73.24	69.18	67.77
Network features in all layers	92.59	91.93	91.51	90.91	93.27	87.18	91.49
ROI features in the 4th layer	88.69	85.63	87.72	87.65	86.74	77.38	88.77
ROI features and network features in the 4th layer	94.41	95.58	94.42	93.41	92.47	92.82	92.64
Multilevel (ROI features in the 4th layer and network features in all layers)	97.26	99.88	97.27	97.41	97.12	94.53	97.27

ACC: accuracy; AUC: area under receiver operating characteristic curve; SEN: sensitivity; SPE: specificity; *Y*: Youden’s index; *F*: *F*-score; BAC: balanced accuracy

### Weight coefficient

**Fig. 2:**
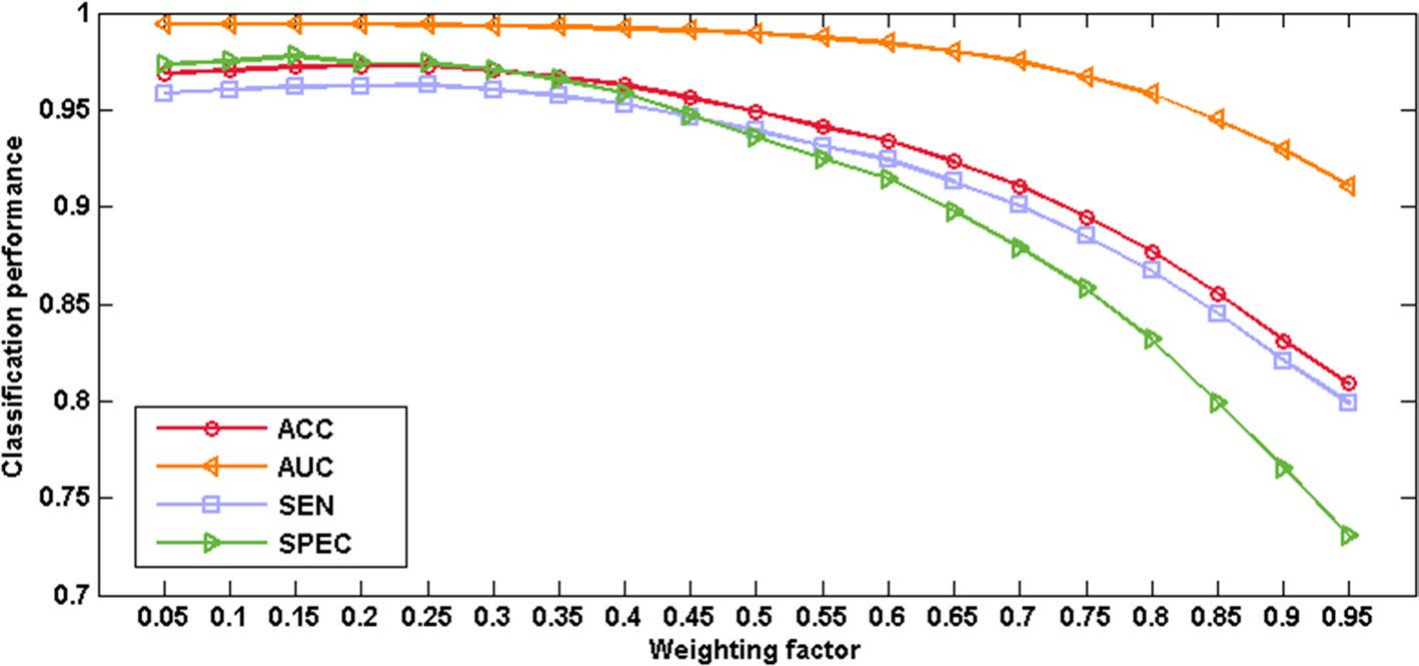
Classification performance with ROI features using different weighting factors. The weight for the ROI features decrease from left to right (range from 0 to 1)

The role of the weight coefficient is to determine the proportion of the various types of features in the multi-kernel classifier (Fig. 2). Appropriate weight coefficient helps achieve the best classifier performance. A smaller weight coefficient indicates that the contribution of ROI features is lower, while the contribution of the hierarchical brain network features is higher. Through experiments, we can find the most suitable weight coefficient in the range of 0–1.

The weight coefficient has an important influence on the performance of the classifier. It is proved that the weight coefficient makes the classifier perform well in the relatively large range from 0.05 to 0.35, which can decline the difficulty of determining the ratio of the two types of features, which reflects the robustness of our proposed method. The best results are obtained at 0.05. At this time, the hierarchical brain network features contributed more to the classification than the ROI features. This is because the hierarchical brain network can fully express the differences in brain structure between the high self-esteem group and low self-esteem group.

### Top discriminative features

**Fig. 3:**
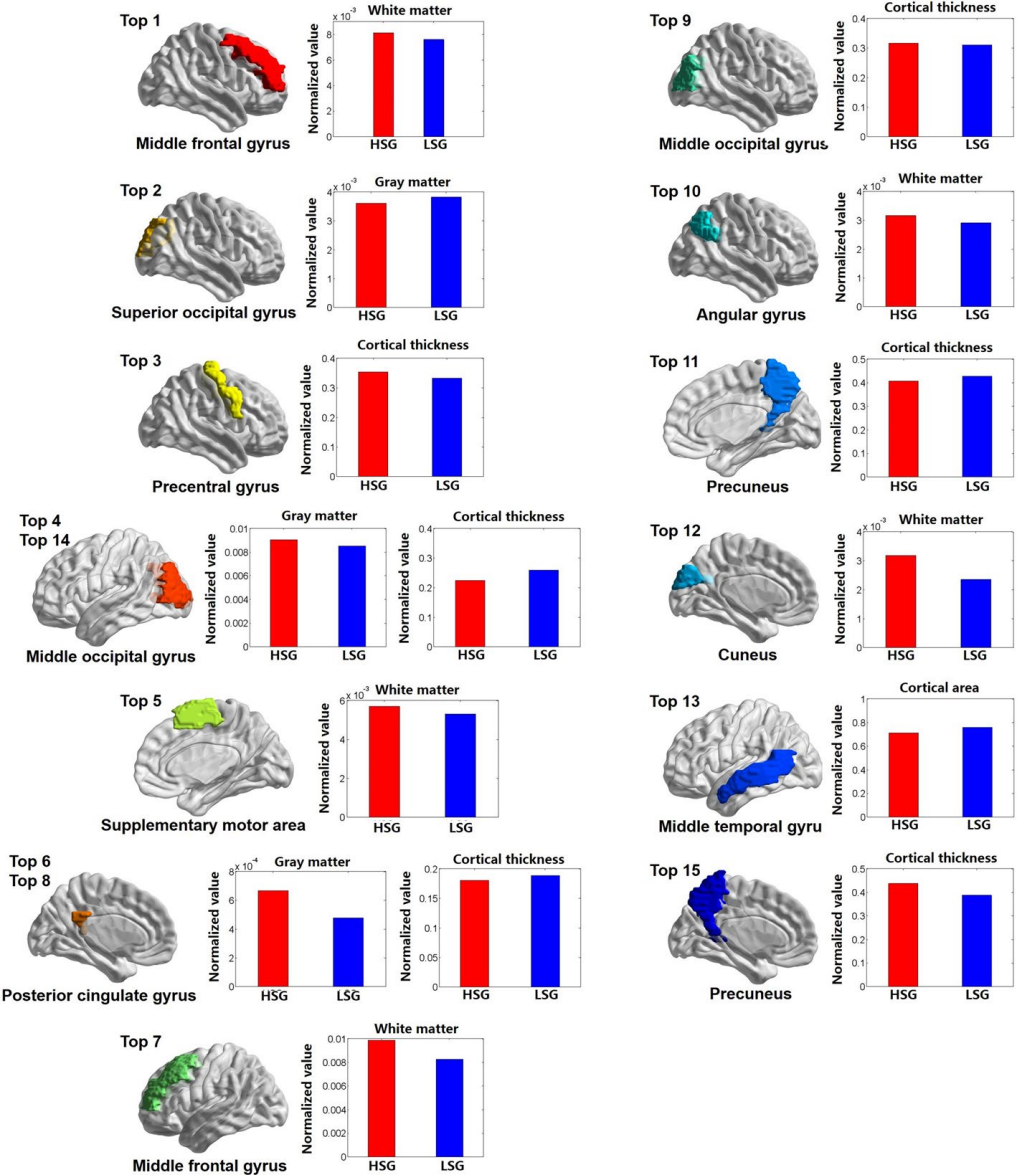
The most discriminating ROI features projected onto the cortical surface. HSG means high self-esteem group and LSG means low self-esteem group

We use the proposed method to select the most discriminative ROI features (Fig. 3). These ROIs include occipital lobe (superior and middle occipital gyrus, cuneus), frontal lobe (supplementary motor area, middle frontal gyrus), temporal lobe (middle temporal gyrus), parietal lobe (precuneus, angular gyrus), limbic lobe (posterior cingulate gyrus), and central region (precentral gyrus). The experimental results also show that differences in brain structure related to self-esteem are mainly in white matter and cortical thickness (Table 2).

**Table 2:** Top 15 most discriminating regional features that were selected using the proposed classification framework

No.	Name of ROI	L/R	Tissue	Brain lobe	Frequency
1	Middle frontal gyrus	R	WM	Frontal lobe	185
2	Superior occipital gyrus	R	GM	Occipital lobe	144
3	Precentral gyrus	R	Thickness	Central region	141
4	Middle occipital gyrus	L	GM	Occipital lobe	102
5	Supplementary motor area	R	WM	Frontal lobe	86
6	Posterior cingulate gyrus	L	CSF	Limbic lobe	75
7	Middle frontal gyrus	L	WM	Frontal lobe	73
8	Posterior cingulate gyrus	L	Thickness	Limbic lobe	70
9	Middle occipital gyrus	R	Thickness	Occipital lobe	68
10	Angular gyrus	R	WM	Parietal lobe	64
11	Precuneus	R	Thickness	Parietal lobe	58
12	Cuneus	L	WM	Occipital lobe	58
13	Middle temporal gyrus	L	Area	Temporal lobe	54
14	Precuneus	L	Thickness	Parietal lobe	53
15	Middle occipital gyrus	L	Thickness	Occipital lobe	53

L: left hemisphere; R: right hemisphere; GM: gray matter volume; WM: white matter volume; CSF: cerebrospinal volume; Thickness: cortical thickness; Area: cortical surface area; Frequency: selected frequency over 100 repetitions of twofold cross-validation

**Fig. 4:**
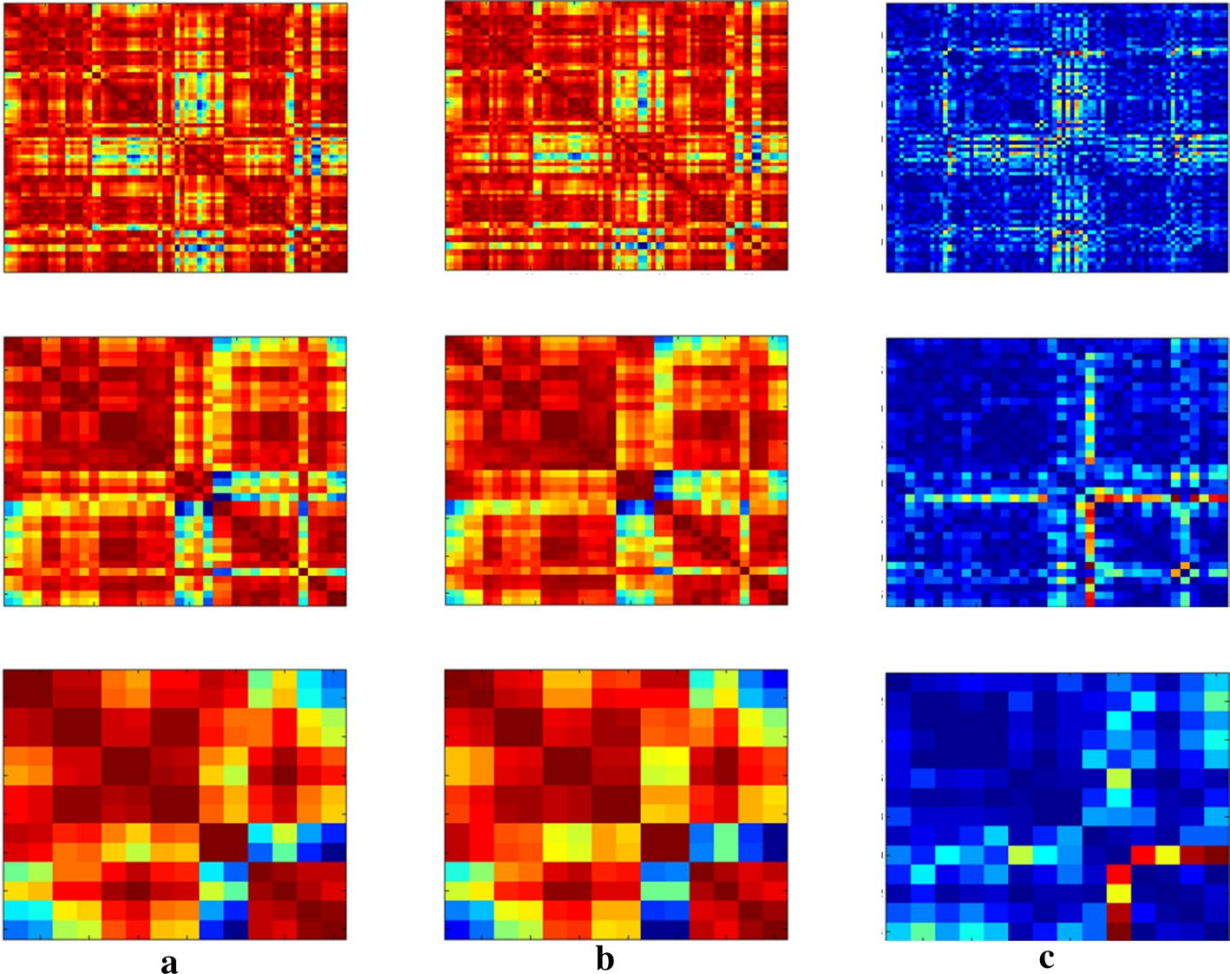
Correlative matrix: **a** high self-esteem group, b low self-esteem group, **c** differences between the two groups

The top 15 network features selected from all four layers (Table 3). The most discriminative hierarchical network features are mainly distributed in limbic lobe and parietal lobe (Fig. 4).

**Table 3:** Top 15 similarity features that were selected using the proposed classification framework

Network	Name of ROI	L/R	Name of ROI	L/R	No.	Frequency
Network 4	Orbitofrontal cortex (inferior)	L	Superior parietal gyrus	L	15	45
	Rectus gyrus	L	Precuneus	L	12	48
	Orbitofrontal cortex (inferior)	L	Paracentral lobule	R	10	54
	Orbitofrontal cortex (inferior)	R	Precuneus	L	3	95
Network 3	Parietal lobe: lateral surface	R	Limbic lobe: Temporal pole (superior)	R	1	118
	Frontal lobe: lateral surface	L	Parietal lobe: lateral surface	L	2	114
	Temporal lobe: lateral surface	L	Parietal lobe: lateral surface	R	4	93
	Frontal lobe: lateral surface	R	Temporal lobe: lateral surface	R	5	93
	Frontal lobe: lateral surface	R	Parietal lobe: lateral surface	L	6	92
	Central region: Rolandic operculum	L	Limbic lobe: temporal pole (superior)	L	7	74
	Central region: postcentral gyrus	R	Parietal lobe: lateral surface	R	9	61
	Temporal lobe: lateral surface	R	Limbic lobe: temporal pole (superior)	R	13	47
	Parietal lobe: lateral surface	L	Limbic lobe: temporal pole (superior)	L	14	47
Network 2	Central region	L	Limbic lobe	L	8	73
	Central region	R	Limbic lobe	L	11	54

L: left hemisphere; R: right hemisphere; Frequency: selected frequency over 100 repetitions of twofold cross-validation

## Discussion

We studied multiple anatomical brain network related to self-esteem. Our results demonstrate that the proposed method is superior to the single-layer network method. The multiple networks enhance the representation of the specific brain structure related to self-esteem, thereby providing an effective and novel method to detect self-esteem-related biomarkers.

### Improvement of the proposed method

It is difficult to fully understand the functional organization of the brain using only a single-layer network framework since the brain is a complex system. In this study, we construct a multiple anatomical brain network in multi-resolution ROIs to improve the classification performance. Compared with the single-layer network-based method, multiple networks enhance the classification performance by using supplementary information from different networks. Compared with the best results obtained using a single-layer network, our proposed method can improve the classification accuracy by 8.95% (Table 1).

### Analysis of discriminative features

The discriminative ROI features discovered by our method are distributed in multiple regions of the brain. Because few current studies employ automatic classification method to study the brain structure of self-esteem, we only compare brain regions found through our machine learning method with existing morphological based studies. Compared with previous studies, our results showed consistency in departmental brain regions, including precuneus [4], precentral gyrus [15], middle frontal gyrus [16], cuneus [4], posterior cingulate [17]. This indicates the effectiveness of our classification method in revealing brain regions related to self-esteem. In addition to these consistent regions, we also found that the middle occipital, superior occipital, and supplementary motor are related to self-esteem. These brain regions have not been reported in previous studies, which is worth paying attention to in the follow-up study.

The discriminative network features are mainly located on frontal, parietal and limbic lobe. After a comprehensive analysis of existing research on neuropsychological mechanisms related to self-esteem, we found that the frontal region is an important part of the neural basis related to self-esteem. The frontal lobe is responsible for self-evaluation, self-regulation, and emotion management. Individuals with low self-esteem have a stronger emotional response to social evaluations, while high self-esteem individuals show stronger self-positivity in the process of self-evaluation. These findings indicate that frontal lobe plays an important role in generating positive self-information.

### Comparison with other methods

Since few studies have used machine learning to analyze the relationship between self-esteem and brain structure, we compare our results with the current morphological studies related to self-esteem. At present, most studies have found a correlation between self-esteem and frontal lobe [6, 16, 17]. The frontal lobe is mainly responsible for the cognitive activities of the brain, and self-esteem involves cognitive processing and emotional response. The results of this paper are generally consistent with those of previous studies. In addition, some specific brain regions, such as the cuneus, have been found to be related to many self-related functions, such as self-related information processing and various aspects of consciousness [16]. In addition, we also found a correlation between cingulate cortex and self-esteem. Studies have shown that when individuals are accepted by society, individuals activate the ventral anterior cingulate cortex and medial prefrontal cortex, thus enhancing self-esteem [17]. Therefore, the study of brain structure with different self-esteem grades is helpful to understand the neurophysiological mechanism of self-esteem.

At the same time, in order to prove the effectiveness of the proposed method. We have compared the classification performance with different classifiers. Table 4 shows the classification results using different classifies. The results show that the proposed method based on multiple anatomical brain networks has better performance than all the other classifiers.

**Table 4:** The classification results using different classifies

Configurations	Classification accuracy (%)
SVM (RBF)	97.27
SVM (linear)	97.07
K-nearest neighbor classifier	94.25
Naive Bayes classifier	92.13
Decision-tree algorithm	91.19

### Limitations and future directions

Although the classification performance is good, our study still has some limitations. Here, we put forward some future directions in order to conduct a better research on self-esteem in cognitive neuroscience. First, as a preliminary study, we use relatively small amount of data in machine learning. In the follow-up study, we will collect some more data to get more reasonable analysis and make more detailed grades of self-esteem, taking psychological, individual, and social factors into account. Second, other metrics for modeling the interactions between ROIs, such as Euclidean distance and the *L*1-norm distance measure. Third, with the increasing of the data, deep learning can also be used to automatically extract features to find the discriminative features of multiple brain network, such as depth automatic encoder. Fourth, due to the multi-types of features involved in this study, multiple weight factors can be used for better feature fusion.

## Conclusion

In this study, we have presented how multiple anatomical brain networks can be used to analyze brain structural differences among undergraduates with different grades of self-esteem. Several feature selection methods are adopted to reduce the number of features, and multi-kernel SVM was employed to integrate various types of features by appropriate weight coefficient. The features extracted from these networks can be used to improve the defects that the traditional single-layer brain network contains insufficient information. The experiments show that our method has improved performance compared with the single-layer network structure, which can provide a new perspective for the analysis of brain structure differences of self-esteem. It also has potential guiding significance of out method in other researches involved in brain cognitive activity and brain disease diagnosis.

## Methods

### Subjects

The structural MRI data used in our study were acquired from Soochow University, which is composed of 68 undergraduates. The study was approved by the Ethics Committee of the Third Affiliated Hospital of Soochow University. Written informed consents were obtained from all subjects. All subjects did not receive stimulants or hypnotics before acquisition in order to keep them awake and let the brain work normally. All participants' vision were normal or corrected to normal, and they were right-handed. After the test, each participant will receive a small gift or financial reward. All subjects are required to perform Rosenberg Self-esteem Scale (RSES) test. The RSES is originally developed by Rosenberg in 1965 to assess the overall feelings of undergraduates about self-worth and self-acceptance. It is the most used self-esteem measurement tool in the psychology community [18]. We ranked the RSES test scores from highest to lowest, and then divided them into two groups: high self-esteem group and low self-esteem group. Table 5 provides detailed information of all participants.

**Table 5:** Demographic information of all subjects

	High self-esteem group	Low self-esteem group	*p* value
Subjects	34	34	
Male/female	19/15	16/18	0.83
Age (mean ± SD)	21.90 ± 1.16	22.53 ± 1.42	0.77
Rosenberg Scale (mean ± SD)	25.35 ± 0.81	17.86 ± 3.35	< 0.001

The *p*-value of gender was obtained by Chi-squared test

The *p*-values of age and Rosenberg scale were obtained by *t*-test

Significance level was set to 0.05

### Imaging acquisition and preprocessing

All images were collected on a 3T Siemens Medical Systems equipment. The acquisition parameters are set as: echo time (TE) = 2.98 ms, repetition time (TR) = 2300 ms, flip angle (FA) = 9 °, voxel size = 1 × 1 × 1 mm^3^, slice thickness = 1 mm, field of view (FoV) = 256 mm.

We use an automatic pipeline for sMRI image processing. Firstly, we adjusted the image orientation (axial, coronal, and sagittal) to match the template image, and performed offset field correction to remove the gray-scale unevenness of the image [19]. Secondly, the brain image was extracted by removing the skull and cerebellum [20]. Thirdly, gray matter (GM), white matter (WM) and cerebrospinal fluid (CSF) were segmented from the background [21]. Fourth, the segmented image was registered to the template labeled with the Automated Anatomical Labeling (AAL) template [22]. Fifth, in order to calculate the morphological features based on the cortex, the middle layer of the cerebral cortex was depicted [23]. After the whole processing, the morphological measurements of GM volume, WM volume, CSF volume, cortical thickness, and cortical surface area of each ROI were obtained for each subject. It should be noted that we removed 12 subcortical ROIs from AAL template considering that the cerebral cortex contains more neurons.

### Framework of the proposed method

**Fig. 5:**
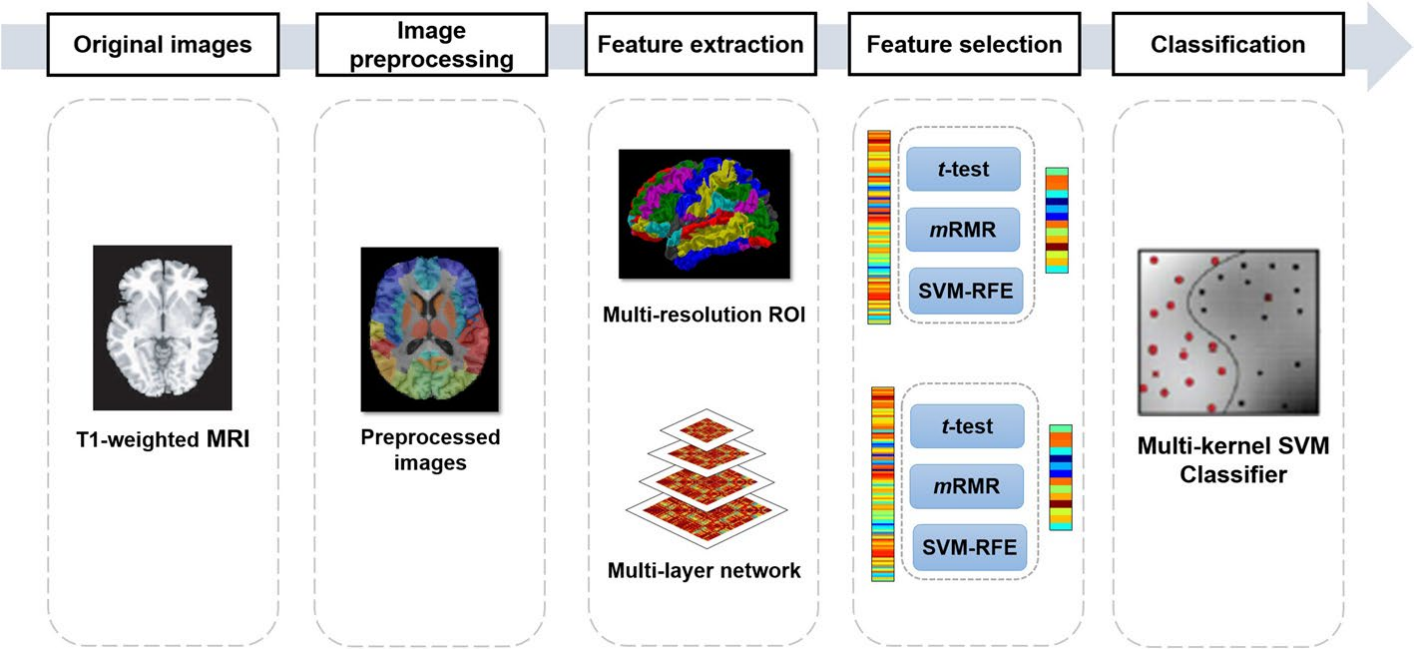
Framework of the classification method using multilevel network features

The framework of the proposed method based on multiple brain network is shown in Fig. 5, mainly including image processing, feature extraction, feature selection, and classification. Multiple brain network were constructed based on morphological features (volume of different brain tissue, cortical thickness, and cortical surface area). Feature selection can reduce the dimensionality of high-dimensional brain network features, only retaining the features that can maximize the specificity of the subjects. The optimal feature subset can be trained by the classifier as neuroimaging markers representing different self-esteem levels.

### Construction of multiple anatomical networks

Through the above image processing steps, GM volume, WM volume, CSF volume, cortical thickness, and cortical surface area of each ROI can be obtained from the MRI image of each subject. In order to reduce individual differences, standardization was performed, dividing the measured value of each ROI by the total intracranial volume, mean cortical thickness, and whole cerebral cortical surface area of the subject. Therefore, we used normalized volume and cortical features to provide a more appropriate representation. More objective measurements can be received by such processing. In order to improve the performance of the classifier, we propose a four-layer hierarchical network in this paper. We used brain templates with different ROI resolution in each layer to construct brain network nodes and edges.

Specifically, the bottommost template containing 78 ROIs is defined as $L^{4}$, the remaining three layers are defined as $L^{l}$, where $l = 1,2,3$. A larger $l$ value indicates a higher-resolution ROI, which is located in the brain network layer closer to the bottom of the hierarchy. By merging small brain regions into large brain functional areas, the number of ROIs is reduced. In the layer $L^{3}$, there are 36 ROIs by dividing the whole brain into lateral, medial and inferior surfaces. In the layer $L^{2}$, 14 ROIs are defined reefing to the anatomical brain structure of central, frontal, parietal, occipital, temporal, limbic, and insula lobe. The specific definition rules of these ROIs can be found in Table 6. It is worth noting that in the first layer $L^{1}$, we study the brain as a whole.

**Table 6:** Regions of interest (ROIs) defined in the automated anatomical labeling (AAL) template

Network 2		Network 3		Network 4	
No.	Name of ROI	No.	Name of ROI	No.	Name of ROI
1, 2	Central region	1, 2	Central region: precentral gyrus	1, 2	Precentral gyrus
		3, 4	Central region: postcentral gyrus	53, 54	Postcentral gyrus
		5, 6	Central region: Rolandic operculum	17, 18	Rolandic operculum left
3, 4	Frontal lobe	7, 8	Frontal lobe: lateral surface	3, 4	Superior frontal gyrus (dorsal)
				7, 8	Middle frontal gyrus
				11, 12	Inferior frontal gyrus (opercular)
				13, 14	Inferior frontal gyrus (triangular)
		9, 10	Frontal lobe: medial surface	19, 20	Supplementary motor area
				23, 24	Superior frontal gyrus (medial)
				65, 66	Paracentral lobule
		11, 12	Frontal lobe: orbital surface	5, 6	Orbitofrontal cortex (superior)
				9, 10	Orbitofrontal cortex (middle)
				15, 16	Orbitofrontal cortex (inferior)
				21, 22	Olfactory
				25, 26	Orbitofrontal cortex (medial)
				27, 28	Rectus gyrus
5, 6	Temporal lobe	13, 14	Temporal lobe: lateral surface	67, 68	Heschl gyrus
				69, 70	Superior temporal gyrus
				73, 74	Middle temporal gyrus
				77, 78	Inferior temporal gyrus
7, 8	Parietal lobe	15, 16	Parietal lobe: lateral surface	55, 56	Superior parietal gyrus
				57, 58	Inferior parietal lobule
				59, 60	Supramarginal gyrus
				61, 62	Angular gyrus
		17, 18	Parietal lobe: medial surface	63, 64	Precuneus
9, 10	Occipital lobe	19, 20	Occipital lobe: lateral surface	45, 46	Superior occipital gyrus
				47, 48	Middle occipital gyrus
				49, 50	Inferior occipital gyrus
		21, 22	Occipital lobe: medial and inferior surfaces	39, 40	Calcarine cortex
				41, 42	Cuneus
				43, 44	Lingual gyrus
				51, 52	Fusiform gyrus
11, 12	Limbic lobe	23, 24	Limbic lobe: temporal pole (superior)	71, 72	Temporal pole (superior)
		25, 26	Limbic lobe: temporal pole (middle)	75, 76	Temporal pole (middle)
		27, 28	Limbic lobe: anterior cingulate gyrus	31, 32	Anterior cingulate gyrus
		29, 30	Limbic lobe: middle cingulate gyrus	33, 34	Middle cingulate gyrus
		31, 32	Limbic lobe: posterior cingulate gyrus	35, 36	Posterior cingulate gyrus
		33, 34	Limbic lobe: Parahippocampal gyrus	37,38	Parahippocampal gyrus
13, 14	Insula	35, 36	Insula: insula	29, 30	Insula

For each layer, correlation between ROIs can be calculated using brain template defined above. Its node correspond to the ROIs in different resolution, and the edge corresponds to the interaction between pairs of ROIs. Take the bottom layer $L^{4}$ as an example, a 78 $\times$ 78 matrix $C^{4}$ can be calculated by computing the Pearson correlation coefficient between the *i*th ROI and *j*th ROI. We define

1 $$C^{4} \left( {i,j} \right) = \exp \left\{ { - \frac{{\left[ {t\left( i \right) - t\left( j \right)} \right]^{2} }}{{2\sigma^{2} }}} \right\} ,$$

where $t\left(i\right)$ and $t(j)$ represent the mean thickness of the cerebral cortex corresponding to the $i$th and $j$th ROIs.

$\sigma$ is defined as $\sigma =\sqrt{{{\sigma }_{i}}^{2}+{{\sigma }_{j}}^{2}}$, where ${\sigma }_{i}$ and ${\sigma }_{j}$ represent the standard deviation of cortex for the $i$th and $j$th ROI. Due to the symmetry of the correlation matrix, we only use the upper triangular elements of the matrix $C^{4}$ to construct the feature vector. We connect the 3003 upper triangular elements to form the corresponding feature vector for $L^{4}$. Since the ROIs in the remaining three layers are obtained by merging ROIs in the bottommost layer, the mean and standard deviation of these compound ROIs can be obtained by calculating the average value of all ROIs. The definition of correlation matrix $C^{l}$ for other layers is similar to $C^{4}$. The union of the hierarchical networks is constructed by junction of the four upper triangular correlation matrix into a long vector.

### Feature selection

In order to reduce the feature dimension and filter out the most discriminative features, we adopted several feature selection methods. First, we preliminarily select the features by comparing the statistics of different features. The statistical *t*-test (*p* < 0.05) is adopted to remove features with small differences (the features with small differences are difficult to distinguish the two groups). Then, another filter-based feature selection method called minimum redundancy and maximum correlation (mRMR) is used to remove the redundant features [24]. The core idea of mRMR is to maximize the correlation between features and classification variables, and minimize the correlation between different features. After the above two filter-based feature selections, the machine learning recursive feature elimination (SVM-RFE) method [25] is used to further reduce the feature dimension. SVM-RFE is proposed in classification of cancer, and has good performance and strong generalization ability. It is the combination of SVM and subsequent search strategy. It trains samples through the model, and then ranks the scores of each feature to remove the feature with the smallest score, and then trains the model again with the remaining features for the next iteration, and finally selects the number of features that are needed. After completing the entire feature selection steps, the optimal feature subset is obtained.

### Classification using multi-kernel SVM

There are various types of features in the multiple brain network, one is the high-resolution ROI features in the fourth layer, and the other is the brain network features corresponding to different layers. Multi-kernel machine learning method can integrate these various types of features into a final classifier. Firstly, a Gaussian Radial Basis Function (RBF) kernel function is used to construct a kernel matrix for each type of feature. Secondly, the two kernel matrices are integrated into the multi-kernel matrix through appropriate weight coefficients [25]. Comparing the results of using linear kernel function and using RBF function (non-linear), we discover that the RBF kernel can significantly improve the classification performance. Therefore, we choose the RBF kernel function to construct the multi-kernel classifier. Finally, the optimal features subset can be obtained.

### Cross-validation

The nested cross-validation method has been applied in our previous research [26]. In the inner loop, the training set are used to determine the parameters of the classifier. In the outer loop, the testing set is used to evaluate the generalization ability of the classifier. It should be noted that at the beginning of the experiment, the entire data set was randomly divided into two parts, one for training and the other one for testing. The training set and testing set can be exchanged throughout the verification process, while the processing steps remain unchanged.

## Data Availability

The datasets used and/or analyzed during the current study are available from the corresponding author on reasonable request.

## Abbreviations

ROI: Region of interest
fMRI: Functional magnetic resonance imaging
sMRI: Structural magnetic resonance imaging
RSES: Rosenberg Self-esteem Scale
TE: Echo time
TR: Repetition time
FA: Flip angle
FoV: Field of view
GM: Gray matter
WM: White matter
CSF: Cerebrospinal fluid
AAL: Automated Anatomical Labeling
mRMR: The minimum redundancy and maximum correlation
SVM-RFE: The machine learning recursive feature elimination
RBF: Radial Basis Function

## References

[1] Saiphoo AN, Halevi LD, Vahedi Z (2020). Social networking site use and self-esteem: a meta-analytic review. Personal Individ Differ.

[2] Rieger S, Göllner R, Trautwein U (2016). Low self-esteem prospectively predicts depression in the transition to young adulthood: a replication of Orth, Robins, and Roberts. J Pers Soc Psychol.

[3] Chavez RS, Heatherton TF (2017). Structural integrity of frontostriatal connections predicts longitudinal changes in self-esteem. Soc Neurosci.

[4] Zilverstand A, Huang AS, Alia-Klein N (2018). Neuroimaging impaired response inhibition and salience attribution in human drug addiction: a systematic review. Neuron.

[5] Wang Y, Zhang L, Kong X (2016). Pathway to neural resilience: Self-esteem buffers against deleterious effects of poverty on the hippocampus. Hum Brain Mapp.

[6] Lieberman MD, Straccia MA, Meyer ML (2019). Social, self,(situational), and affective processes in medial prefrontal cortex (MPFC): Causal, multivariate, and reverse inference evidence. Neurosci Biobehav Rev.

[7] Bede P, Hardiman O (2018). Longitudinal structural changes in ALS: a three time-point imaging study of white and gray matter degeneration. Amyotroph Later Scler Frontotemporal Degener.

[8] Solé-Casals J, Serra-Grabulosa JM, Romero-Garcia R (2019). Structural brain network of gifted children has a more integrated and versatile topology. Brain Struct Funct.

[9] Kelley WM, Macrae CN, Wyland CL (2002). Finding the self? An event-related fMRI study. J Cogn Neurosci.

[10] Goldin P, Ziv M, Jazaieri H (2012). Randomized controlled trial of mindfulness-based stress reduction versus aerobic exercise: effects on the self-referential brain network in social anxiety disorder. Front Hum Neurosci.

[11] Cuingnet R, Gerardin E, Tessieras J (2011). Automatic classification of patients with Alzheimer's disease from structural MRI: a comparison of ten methods using the ADNI database. Neuroimage.

[12] Schmitgen MM, Niedtfeld I, Schmitt R (2019). Individualized treatment response prediction of dialectical behavior therapy for borderline personality disorder using multimodal magnetic resonance imaging. Brain Behav.

[13] Erol RY, Orth U (2011). Self-esteem development from age 14 to 30 years: a longitudinal study. J Pers Soc Psychol.

[14] Peng B, Saxena A, Wang S, et al. Enhancing the representation of multiple anatomical network for young adults with self-esteem difference. In: 2019 12th international congress on image and signal processing, BioMedical Engineering and Informatics (CISP-BMEI). IEEE. 2019;1–5.

[15] Cheng W, Rolls ET, Qiu J (2018). Functional connectivity of the precuneus in unmedicated patients with depression. Biol Psychiatry Cogn Neurosci Neuroimaging.

[16] Yang J, Xu X, Chen Y (2016). Trait self-esteem and neural activities related to self-evaluation and social feedback. Sci Rep.

[17] Van Schie CC, Chiu CD, Rombouts SARB (2018). When compliments do not hit but critiques do: an fMRI study into self-esteem and self-knowledge in processing social feedback. Soc Cogn Affect Neurosci.

[18] García JA, y Olmos FC, Matheu ML (2019). Self esteem levels vs global scores on the Rosenberg self-esteem scale. Heliyon.

[19] Thirion JP (1998). Image matching as a diffusion process: an analogy with Maxwell's demons. Med Image Anal..

[20] Smith SM (2002). Fast robust automated brain extraction. Hum Brain Mapp.

[21] Wang L, Shi F, Li G (2013). 4D segmentation of brain MR images with constrained cortical thickness variation. PLoS ONE.

[22] Tzourio-Mazoyer N, Landeau B, Papathanassiou D (2002). Automated anatomical labeling of activations in SPM using a macroscopic anatomical parcellation of the MNI MRI single-subject brain. Neuroimage.

[23] Li G, Wang L, Yap PT (2019). Computational neuroanatomy of baby brains: a review. NeuroImage.

[24] Peng H, Xie P, Liu L, et al. Brain-wide single neuron reconstruction reveals morphological diversity in molecularly defined striatal, thalamic, cortical and claustral neuron types. bioRxiv. 2020;675280.

[25] Guyon I, Weston J, Barnhill S (2002). Gene selection for cancer classification using support vector machines. Mach Learn.

[26] Peng B, Lu J, Saxena A (2017). Examining brain morphometry associated with self-esteem in young adults using multilevel-ROI-features-based classification method. Front Comput Neurosci.

